# Air Traffic Controller Workload Detection Based on EEG Signals

**DOI:** 10.3390/s24165301

**Published:** 2024-08-15

**Authors:** Quan Shao, Hui Li, Zhe Sun

**Affiliations:** College of Civil Aviation, Nanjing University of Aeronautics and Astronautics, Nanjing 211106, China; lihui123@nuaa.edu.cn (H.L.); sx2207075@nuaa.edu.cn (Z.S.)

**Keywords:** air traffic controller, Electroencephalogram (EEG), machine learning, workload

## Abstract

The assessment of the cognitive workload experienced by air traffic controllers is a complex and prominent issue in the research community. This study introduces new indicators related to gamma waves to detect controllers’ workload and develops experimental protocols to capture their EEG data and NASA-TXL data. Then, statistical tests, including the Shapiro–Wilk test and ANOVA, were used to verify whether there was a significant difference between the workload data of the controllers in different scenarios. Furthermore, the Support Vector Machine (SVM) classifier was employed to assess the detection accuracy of these indicators across four categorizations. According to the outcomes, hypotheses suggesting a strong correlation between gamma waves and an air traffic controller’s workload were put forward and subsequently verified; meanwhile, compared with traditional indicators, the indicators associated with gamma waves proposed in this paper have higher accuracy. In addition, to explore the applicability of the indicator, sensitive channels were selected based on the mRMR algorithm for the indicator with the highest accuracy, β + θ + α + γ, showcasing a recognition rate of a single channel exceeding 95% of the full channel, which meets the requirements of convenience and accuracy in practical applications. In conclusion, this study demonstrates that utilizing EEG gamma wave-associated indicators can offer valuable insights into analyzing workload levels among air traffic controllers.

## 1. Introduction

The effects of prolonged, high-intensity mental activities on brain fatigue are well-documented, with ramifications on mood and physical function [1]. Particularly in the case of air traffic controllers, mental fatigue can significantly impair cognitive abilities, reaction times, and overall alertness, posing significant implications for operational performance or even leading to safety incidents [2,3,4,5]. As air traffic controllers play a crucial role in civil aviation, the detection and analysis of their workload are of the utmost importance, particularly given the increasing volume of flights [6].

The current approaches to workload detection for air traffic controllers can be broadly categorized into subjective and objective methods. Subjective methods, such as the KSS scale and NASA-TLX scale, are relatively easier to implement and more widely applicable, but frequent completion of these scales may disrupt experimental subjects and add to their workload [7,8,9]. On the other hand, objective methods involving the analysis of physiological signals, such as EEG and ECG signal detection [10], eye and facial feature detection [11], and voice feature detection [12], offer high reliability and validity, accurately capturing real-time physiological changes. When comparing the two method types, subjective detection methods are relatively easier to implement, faster, and more widely applicable. 

Notably, the high temporal resolution and portability of EEG signals have established their credibility as a dependable tool for evaluating the workload of air traffic controllers, making them an essential technical resource for assessing workload in this field [12,13,14]. In addition, studies have underscored the sensitivity of EEG to vigilance fluctuations and its ability to forecast performance decline resulting from sustained mental exertion [15,16]. Consequently, in this study, EEG signals will be utilized to investigate the workload of controllers.

Electroencephalography (EEG) measures the brain’s electrical activity, which is recorded on the scalp using a network of regularly spaced electrodes. These signals are categorized into various frequency bands, encompassing delta waves (1–4 Hz), theta waves (4–8 Hz), alpha waves (8–13 Hz), beta waves (13–30 Hz), and gamma waves (above 30 Hz). Delta waves are linked with deep relaxation and restorative sleep, while theta waves are prevalent during trance states or hypnosis. Alpha waves promote a deeper sense of relaxation and contentment, positioned between the conscious (beta) and subconscious (theta) mind. Beta waves represent the most common high-frequency waves, and gamma waves mirror heightened cognitive activity and neuronal excitability [16,17,18,19,20,21]. Overall, the analysis of EEG waveforms and their division into different frequency bands is frequently employed to gauge variations in the “internal” state of subjects during air traffic control simulation tasks [22,23,24,25,26,27].

In 2010, Dasari et al. conducted a study where they continuously monitored the EEG changes of civil aviation controllers during a 2 h simulator test [28]. They noted that theta, alpha, and beta-band EEG waves could effectively depict the mental state transformation of controllers during their tasks. These findings were acknowledged by the FAA. Shou et al. investigated the fluctuations of EEG data during controller tasks and observed that the peak value of theta waves fluctuated significantly with increasing workload, indicating that theta waves may provide a better reflection of workload-related changes [29]. Budi Thomas Jap et al. applied the fast Fourier transform method to analyze four EEG features: (theta + alpha)/beta, alpha/beta, (theta + alpha)/(alpha + beta), and theta/beta. They discovered that alpha wave content increased and beta wave content decreased during severe brain fatigue [30]. Arico et al. analyzed the frontal and occipital theta waves, as well as the parietal alpha waves, of control trainees after completing simulator tests. The results confirmed the reliability of assessing workload over time [13]. Wang et al. utilized theta/(alpha + beta) as an indicator to detect controller fatigue and found that fatigue levels at 0:00 were significantly higher than at 12:00, with the reaction time for abnormal situations showing a linear relationship with the initial fatigue level [31]. Trapsilawati et al. [32] investigated the EEG signals of controllers during conflict handling and found that convergent conflict and cross-conflicts led to decreased situational awareness, increased stress levels, and activation of temporal and parietal theta waves.

In general, the focus of EEG research on air traffic controllers has predominantly centered on characterizing theta, alpha, and beta waves, largely overlooking the potential role of gamma waves. Historically, higher frequency waves were often disregarded as mere noise and systematically filtered for decades, which has contributed to the limited attention on gamma band EEG analysis specifically aimed at air traffic controllers [33]. Although low-frequency gamma waves have been applied to cognitive load [34] and workload [35,36] for air traffic controllers, research on higher-frequency gamma waves remains relatively limited. However, it is important to note that higher-frequency gamma waves are indicative of advanced cognitive processes and hold potential for analyzing the workload of air traffic controllers.

Furthermore, while multitasking is a common occurrence in air traffic control activities, the literature currently lacks clear evidence regarding the EEG’s sensitivity in differentiating various levels of complexity in multitasking conditions [37]. Additionally, the use of a multichannel EEG acquisition system, such as the 64-channel EEG system mentioned in this paper, presents a challenge as it is complex equipment typically found in laboratories or hospitals. It necessitates well-trained technicians to properly place the electrodes, a process that is both time-consuming and labor-intensive. These factors make it difficult to integrate such systems into the daily work of controllers. Therefore, the development of a more practical EEG system with fewer channels, or even a single-channel system, for estimating the workload of controllers is imperative. Such a system should be portable, affordable, simple, and user-friendly in order to effectively support the needs of air traffic controllers.

In considering the aforementioned points, it is essential to acknowledge the significant strides made by researchers in studying controller fatigue using EEG signals. However, it is important to recognize the substantial differences that exist between the airspace delineation environment and operational habits and processes of Chinese controllers when compared with controllers from other nations [38,39]. Consequently, the research findings cannot be directly applied and must be adapted to suit the unique conditions of frontline control units.

To advance the detection and analysis of controllers’ workloads and verify the potential use of gamma waves for workload detection, this study conducted experiments to collect EEG data from controllers. Based on the experiment results, the hypothesis was formulated as follows: Gamma waves can effectively detect changes in controller workload. Subsequently, the study aims to validate the hypothesis by examining the distinguishability of gamma-containing and non-gamma-containing indicators using various categorization methods. The most effective indicator identified will then be utilized for workload detection, with its efficacy assessed based on its detection accuracy across different channel combinations. It is anticipated that through this process, an indicator and a limited number of channels will be identified to enable precise, straightforward, and rapid detection of controllers’ workloads.

## 2. Materials Method

### 2.1. Experimental Design

#### 2.1.1. Subjects and Experimental Environment

The study involved sixteen seasoned controllers, each possessing a minimum of three years of experience in control systems and ranging in age from 27 to 37. In the experiment, a control simulator was employed to manage workload. Participants were engaged in control activities within simulated environments featuring varying degrees of heavy traffic. To gather EEG data, a 64-electrode Borecon NeuSen W-series wireless EEG acquisition system was utilized.

#### 2.1.2. Experimental Scene Selection

The mental workload of air traffic controllers is heavily influenced by the volume of traffic, which is measured by the number of aircraft requiring handling within a specific time frame, including both landings and take-offs. With increased traffic, controllers are tasked with managing a larger number of aircraft simultaneously to ensure their safe and efficient operation in the air and on the ground. This heightened workload entails a wide range of responsibilities, including assigning aircraft to appropriate routes and altitudes, coordinating and monitoring communications, and overseeing traffic flow. These responsibilities demand continuous vigilance over multiple aircraft and the ability to make timely and accurate decisions and instructions, all of which contribute to the mental workload and stress experienced by controllers.

In addition to managing traffic flow, various abnormal situations such as equipment malfunctions, foreign object debris on runways, specific operational demands of airports, and incident management can also significantly impact the workload of air traffic controllers. These diverse scenarios, collectively referred to as abnormal situations, require information processing, decision-making, and tailored guidance to ensure aviation safety, thereby placing additional strain on controllers and subjecting them to heightened psychological stress.

Given the aforementioned factors, a preliminary experiment was conducted to select four distinct and representative exercises on the control simulator before the main experiment. The workload for each exercise was categorized based on changes in traffic levels and the presence or absence of abnormal situations, resulting in classifications as lower load, higher load, overload, and abnormal situation scenes. Moreover, a resting state scene (labeled as Scene 0) was included to monitor the EEG data of controllers with their eyes closed. The specific scene settings are detailed in Table 1. Furthermore, for the purposes of this study, an abnormal situation refers to the presence of foreign objects on the runway, and these labels will be utilized to classify the collected EEG data.

To determine the optimal sequence for each scene, preliminary experiments were conducted to compare the distinguishability of experimental data across different sequences, including “Scene 0–Scene 1–Scene 2–Scene 3–Scene 4”, “Scene 4–Scene 3–Scene 2–Scene 1–Scene 0”, and “Scene 0–Scene 3–Scene 2–Scene 1–Scene 4”. Upon analysis, it was found that the EEG data of the subjects exhibited the greatest distinguishability when presented in the order of “Scene 0–Scene 4–Scene 3–Scene 1–Scene 2”. As a result, subsequent experiments will be carried out in this specific order.

#### 2.1.3. Experimental Procedure

**Figure 1:**
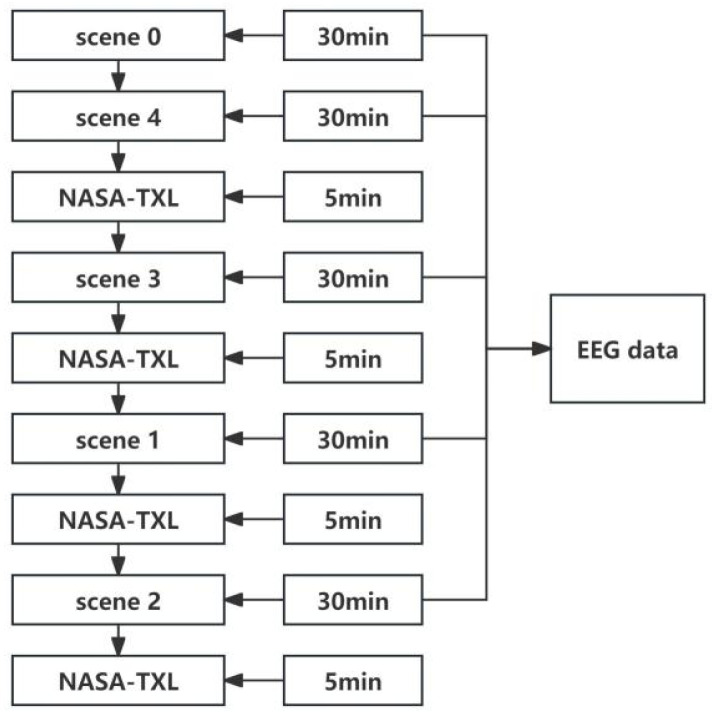
**Experimental Procedure.** Each scene lasted 30 min, and the NASA-TXL scale was filled out about the scene at the end of the scene.

During the experiments, EEG electrodes were positioned in accordance with the International 10–20 System, with a sampling frequency of 1000 Hz. Initially, the EEG data of each subject in the resting state with their eyes closed (scene 0) were collected before simulating the working state. After completing the NASA-TXL scale [40] for this scene, the EEG data during the working state were then recorded. Each scene lasted for half an hour, and an additional five minutes were required to complete the NASA-TXL scale. Subjects were tasked with performing all exercises to ensure the comprehensive collection of intact EEG data and NASA-TXL subjective scale data. The detailed process is illustrated in Figure 1.

### 2.2. Feature Extraction and Classification

After completing the experiment, the NASA-TXL data and EEG data underwent processing for feature extraction and classification. Before analyzing the EEG data, several crucial preprocessing steps were conducted on the collected EEG data. First, unnecessary electrodes, such as ECG, HEOR, HROL, VEOU, etc., were removed, leaving 59 channels available for subsequent analysis.

**Figure 2:**
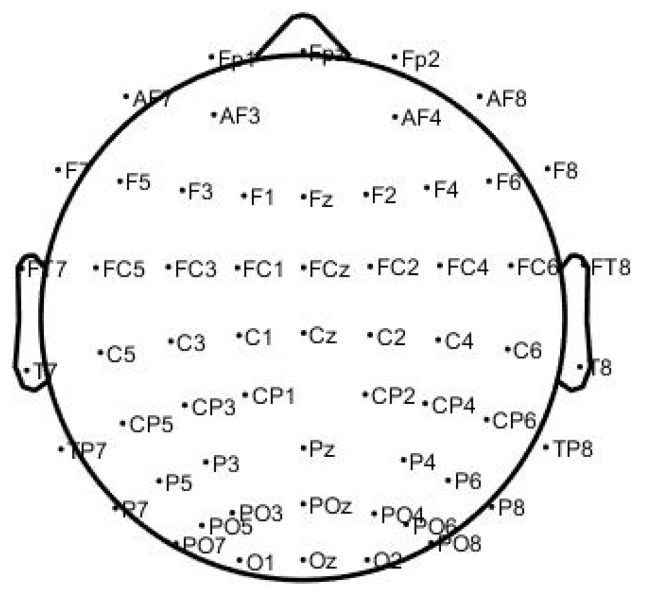
**Channel location by name**.

After completing the experiment, the NASA-TXL data and EEG data underwent processing for feature extraction and classification. Prior to analyzing the EEG data, several essential preprocessing steps were taken to ensure data quality. This involved removing unnecessary electrodes, such as ECG, HEOR, HROL, and VEOU, leaving 59 channels available for subsequent analysis, as shown in Figure 2.

To reduce noise and remove artifacts, a bandpass filter with a frequency range between 0.5 and 100 Hz was applied to each subject’s EEG signal. This filtering step aimed to retain relevant neural activity within desired frequency bands while attenuating noise and out-of-range frequencies.

Regarding re-referencing to the average, we added a zero-filled channel as the initial reference, calculated the average potential, and then subtracted it from all the channels [41]. This way, the smallest eigenvalue of the data (after removing the ‘initial reference’) was about 10^0, which is very large compared with the limit of the effective rank, and ICA can work on full-ranked data. Subsequently, Independent Component Analysis (ICA) was used to decompose the EEG signal and identify and remove potential artifacts or noise components. The informax runica.m algorithm within MATLAB’s EEGLAB toolbox was used with default parameter settings.

Following ICA, additional artifact rejection steps were performed based on visual inspection and statistical criteria to ensure data quality. Any segments of the EEG signal contaminated by excessive noise, movement artifacts, or other disturbances were manually identified and removed from the analysis.

Upon completing these preprocessing steps, the clean EEG data were ready for feature extraction and subsequent classification analyses. The entire preprocessing pipeline was implemented using MATLAB’s EEGLAB, with careful monitoring and validation of all processing steps to ensure data reliability and validity.

Once the preprocessing was completed, the aim was to distinguish changes in brain activity of controllers under different workloads using the EEG power spectrum [42,43]. To achieve this, relevant EEG indicators were extracted and then utilized for classification. Initially, the common traditional indicators were considered, such as α absolute energy, β absolute energy, δ absolute energy, θ absolute energy, α/(δ + β + θ + α), β/(δ + β + θ + α), θ/(δ + β + θ + α), (α + θ)/β, θ/β, α/β, and (α + θ)/(α + β) [30,44]. Additionally, new indicators related to gamma waves with a similar structure to these indicators were proposed for workload analysis, including γ absolute energy, γ/(δ + β + θ + α), γ/(δ + β + θ + α + γ), and so on. All indicators are shown in Table 2, and the first 13 indicators are common working condition detection indicators, while indicators 14–25 related to gamma waves are newly introduced in this study for workload analysis.

After completing the feature extraction, a Pearson correlation analysis was conducted to investigate the relationship between the indicators and workload. The Pearson product–moment correlation coefficient, which measures the correlation between two variables X and Y and provides a value between −1 and 1, was utilized. The calculation formula is as follows:

(1) $$\rho_{X,Y}=\frac{\mathrm{cov}(X,Y)}{\sigma_{X}\sigma_{Y}}=\frac{E((X-\mu_{X})(Y-\mu_{Y}))}{\sigma_{X}\sigma_{Y}}=\frac{E(XY)-E(X)E(Y)}{\sqrt{E(X^{2})E^{2}(X)}\sqrt{E(Y^{2})E^{2}(Y)}}$$

Specifically, −1 indicates a perfect negative correlation, while +1 indicates a perfect correlation. The strength of a variable’s correlation is typically assessed based on the following range of values for the correlation coefficient: 0.8–1.0 indicates a very strong correlation, 0.6–0.8 indicates a strong correlation, 0.4–0.6 indicates a moderate correlation, 0.2–0.4 indicates a weak correlation, and 0.0–0.2 indicates an extremely weak correlation or no correlation [45].

Following the Pearson correlation analysis, a Support Vector Machine (SVM) classifier was utilized to categorize the calculated indicators. SVM, a type of generalized linear classifier, performs binary classification through supervised learning, using a maximum-margin hyperplane as the decision boundary based on learned samples [46,47]. The dataset was randomly divided into a training set (70% of the total sample), testing set (15% of the total sample), and validation set (15% of the total sample) to ensure generalizability.

To enhance the generalizability of the results, the data were categorized in various ways and classified by the SVM classifier, including resting/working, 0/high/medium–low load, 0/low/medium–high load, and 0/low/medium/high load. For the classification method of resting/working, the EEG data of a controller at rest was labeled as “resting”, while the EEG data of a controller at work was labeled as “working”. To achieve a balanced distribution of sample data across the two labels, we conducted data sampling from Scene 1, Scene 2, Scene 3, and Scene 4. Specifically, we randomly selected one-fourth of the data from each work scene to represent the input data associated with the “working” label.

Each feature’s corresponding data were classified using the SVM classifier, and a grid search method was employed to optimize the classification results by identifying the best parameter combinations for each feature under different classification methods. Subsequently, the best-performing feature was chosen based on its classification accuracy across different mental workload categories for controller detection.

### 2.3. Application

The preceding work has enabled the identification of the most suitable indicator for detecting the controller workload. Subsequently, the suitability of this indicator for controller workload detection was validated by assessing its detection accuracy in single-channel, multichannel, and full-channel applications.

This paper utilizes the mRMR algorithm to select a single channel or a combination of channels for the indicator, aiming to enhance its applicability. The mRMR algorithm combines correlation and redundancy between features to identify the most relevant and minimally redundant features [48].

This study employs the mRMR algorithm to select a single channel or a combination of channels for the indicator with the objective of enhancing its applicability. The mRMR algorithm integrates correlation and redundancy between features to identify the most relevant and minimally redundant features [48]. The maximal relevance criterion is calculated as:

(2) $$\max D(S,c),D=\frac{1}{\left|S\right|}\sum_{x_{i}\in S}I(x_{i};c)$$

Here, S represents the set of channels, and c represents the target variable (mental workload), with $x_{i}$ being one of the channels. The minimum redundancy criterion is as follows:

(3) $$\min R(S)=\frac{1}{{\left|S\right|}^{2}}\sum_{x_{i},x_{j}\in S}I(x_{i},x_{j})$$

The redundancy between channels can be calculated by using this formula. The criterion that combines the above two constraints is called “minimal-redundancy–maximal-relevance” (mRMR).

(4) max∅(D,R),∅=D−R

According to this algorithm, the set of “minimal-redundancy–maximal-relevance” channels for each subject corresponding to the best-performing indicator can be selected. Furthermore, the applicability of the indicator can be analyzed based on the detection accuracy in different channels or combinations of channels.

## 3. Results

### 3.1. Data of NASA-TXL

The NASA Task Load Index is a multidimensional assessment of workload developed by NASA’s Ames Research Center. It is assessed on six scales: Mental Demands, Physical Demands, Temporal Demands, Own Performance, Effort, and Frustration.

**Figure 3:**
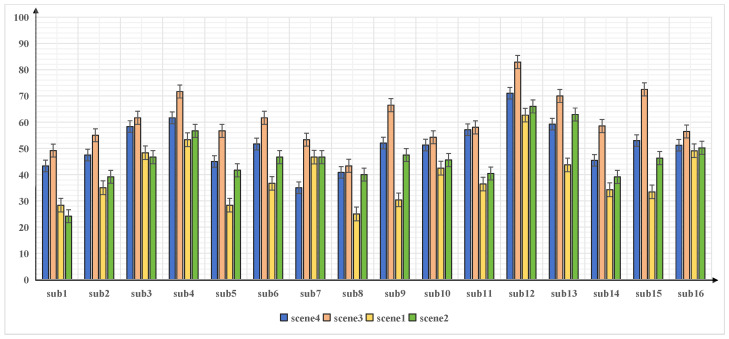
Workload of each sub. Scene 3 always had the highest workload, while Scene 1 always had the lowest workload.

In this study, NASA-TXL scale values were utilized to evaluate workload, gathered from eight subjects rating workload across four different scenes. As illustrated in Figure 3, although individual workload perceptions varied slightly across scenes, a consistent overall trend was observed. Scene 3 consistently exhibited the highest workload, and the scene exhibited nearly the lowest workload, whereas the other scenes indicated lower overall workloads. Moreover, Scene 4 consistently demonstrated a higher workload compared to Scene 2.

Additionally, given that the number of subjects was fewer than 5000, the Shapiro–Wilk normality test was employed to ascertain the data’s validity. If the *p*-value exceeds 0.05 for all four scenes, the data are considered normally distributed, and the scenarios are deemed valid. Otherwise, if the *p*-values are below 0.05, the data are considered invalid, requiring the experiment to be re-evaluated.

The results of the normality test are presented in Table 3. The obtained *p*-values for all four scenes were greater than 0.05, signifying that the NASA-TXL values for the subjects in each scene followed a normal distribution.

Based on the results of the Shapiro–Wilk test, it was confirmed that the workload data of the controllers in each scene followed a normal distribution. Subsequently, a one-way ANOVA was conducted to assess the significance of differences in workload data among the controllers in different scenes. The output indicated an f_statistic value of 13.244017117542056 and a *p*-value of 9.6884759694676 × 10^−7^, with the *p*-value being less than 0.05. This confirms a significant difference in workload data across the various scenarios, thereby validating the selection of the experimental scenes.

### 3.2. Results of Pearson’s Coefficient

**Figure 4:**
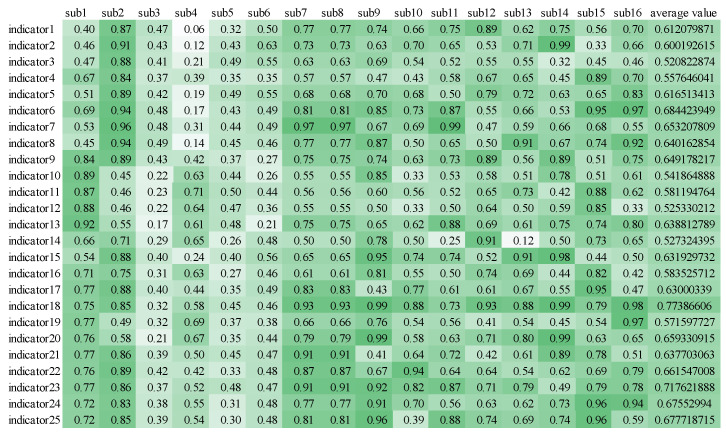
**Pearson’s coefficient heat map.** Darker colors represent greater Pearson correlation coefficients between the indicator and workload. The Pearson’s coefficient of each indicator is greater than 0.5.

Prior to computing Pearson’s coefficient, data processing tasks such as outlier identification and data scaling were performed. The results, depicted in Figure 4 below, reveal that Pearson’s coefficient for each indicator exceeds 0.5. This preliminary finding suggests a correlation between all 25 selected indicators and workload, with 13 indicators demonstrating a strong correlation. These outcomes validate the suitability of all 17 selected indicators for further analysis.

### 3.3. Hypothesis and Verification Base on the Results of Classification

#### 3.3.1. Results of Classification

**Figure 5:**
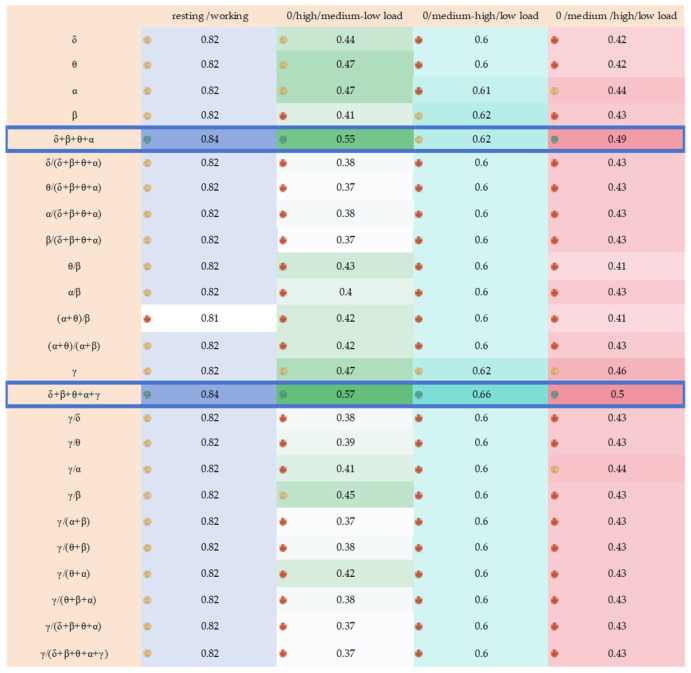
This figure shows the classification results of the indicators under different classification methods; each column represents a classification method, and darker colors of the corresponding data in each column mean a better classification result. In each column of data, red arrows indicate the worst classification accuracy for that classification method, yellow effects indicate moderate classification accuracy, and green arrows indicate the best classification accuracy.

Additionally, the results of the classification process are detailed in Figure 5.

When the data were categorized into resting and working states, all indicators exhibited accuracy above 0.8, affirming the validity of these indicators within this classification.

Furthermore, when the EEG data of controllers are divided into three categories: 0 load, high load (Scene 3 and Scene 4), and medium–low load (Scene 1 and Scene 2), the accuracy of all indicators is above 0.33333. Another way of categorizing involves three categories: 0 load, medium–high load (Scene 2, Scene 3, and Scene 4), and low load (Scene 1), where the accuracy of all indicators reaches 0.6 and above.

The results of the three classifications indicate that the data can be segmented, and the indicators are valid. When the data are divided into four categories: 0 load, medium load (Scene 2), high load (Scene 3 and Scene 4), and low load (Scene 1). The accuracy of all the indicators is much higher than 0.25. The results of the four categories indicate that the data can be effectively classified, and the indicators are valid.

In the classification method of rest/working and 0/low/medium–high workload, the differences in classification accuracy between indicators are minimal. In fact, many indicators have the same classification accuracy, making it difficult to compare the performance of different indicators. In the classifications of 0/low/high workload and 0/low/medium/high workload, indicators show significant differences in classification accuracy. However, the ranking of indicators varies greatly between these two classification methods. Some indicators perform better in the 0/low/high workload classification, while others excel in the 0/low/medium/high workload classification, thereby increasing the complexity of feature selection. In order to have a metric to access the performance of indicators under different classification methods and then enable comparisons between indicators, the data were normalized using the following formula:

(5) $$f(x_{i})=\frac{x_{i}-x_{\min}}{x_{\max}-x_{\min}}$$

**Figure 6:**
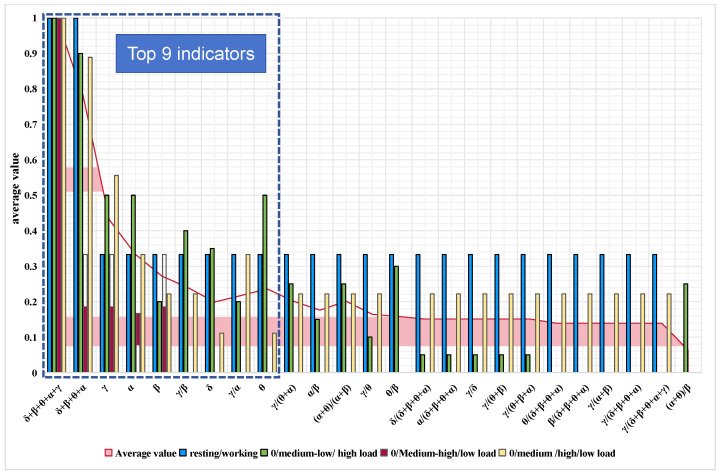
The bar charts show the normalized data for the 25 indicators under different classifications, and the line graphs show the means of the normalized data under different classifications. Indicators are sorted by average value from left to right as the average value goes from highest to lowest. Some indicators correspond to fewer than 4 bars because, in some classifications, f(x_i_) = 0.

$f(x_{i})$ is defined as a value of the indicator after normalization and provides a value between 0 and 1. $f(x_{i})=0$ indicates the lowest accuracy under the corresponding classification method, and $f(x_{i})=1$ means the highest accuracy under the classification method. The normalization results are presented in Figure 6.

It is evident that indicator 15 (δ + β + θ + α + γ) demonstrates a superior categorization ability compared to indicator 5 (δ + β + θ + α) across all categorizations. This suggests that indicators containing gamma waves are more effective in capturing the changes in EEG characteristics resulting from workload variations among air traffic controllers.

Furthermore, among the top nine indicators, with the exception of γ/β and γ/α, all are absolute energy, while the lower-ranked indicators are relative energy. This leads to the speculation that absolute energy may more accurately reflect the workload changes of controllers than relative energy.

Delving into the ranking of absolute energy, it is notable that the absolute energy of δ and θ is ranked lower, while β, α, and γ are ranked higher. This finding implies that controllers are alert during their work, corroborating previous studies [26]. This conclusion further reinforces the reliability of the experimental data.

#### 3.3.2. Hypothesis and Verification

Based on the results above, the following hypothesis is proposed.

**H1:**  *Gamma waves can be used to detect changes in the controller’s workload*.

**H2:**  *Compared with relative energy, absolute energy can better reflect controller workloads*.

To test the hypothesis, new indicators were proposed and processed as described previously. Based on the results, it was found that the absolute energy indicators combined with different frequency rhythms performed better. Consequently, the new indicators comprised absolute energy indicators that combined various frequency rhythms. The results of this analysis, involving a total of 49 indicators organized into 14 groups of gamma-containing and non-gamma-containing indicators, were then normalized and ranked in Table 4.

The ranked indicators reaffirmed the superior effectiveness of absolute energy in reflecting the controllers’ state under varying workloads compared to relative energy. Furthermore, among the 49 indicators, indicator 49 (β + θ + α + γ) emerged with the highest score across all categorizations, making it the most suitable for detecting the workload of controllers.

**Figure 7:**
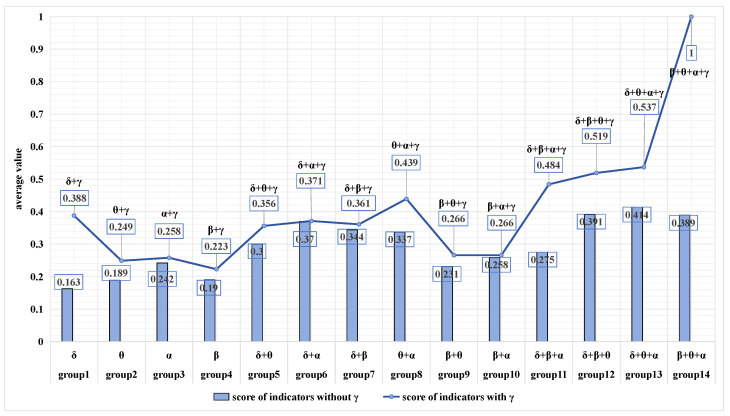
**Comparison chart of control groups.** The bar chart represents the scores of non-gamma-containing indicators, and the line chart represents the scores of gamma-containing indicators. From the figure, we can see that the gamma-containing indicators always have higher scores than the non-gamma-containing indicators.

In order to test hypothesis H1, the indicators with and without gamma were compared, and the results displayed in Figure 7 supported the hypothesis. Across all groups, the gamma-containing indicators consistently scored higher compared to the non-gamma-containing indicators.

Furthermore, to assess whether there is a significant difference between the scores of gamma-containing indicators and non-gamma-containing indicators, a paired samples t-test was implemented on the two data sets. The analysis revealed that the t-statistic yielded a value of −2.7208074278915646, with a corresponding *p*-value of 0.017485943009039176. Given that the *p*-value is less than 0.05, it can be concluded that there is indeed a significant disparity between the two sets of data. This outcome validates the initial hypothesis and supports the conclusion that gamma waves can effectively be utilized to detect the workload of controllers. This validated the hypothesis and led to the conclusion that gamma waves can indeed be utilized to detect the workload of controllers.

**Figure 8:**
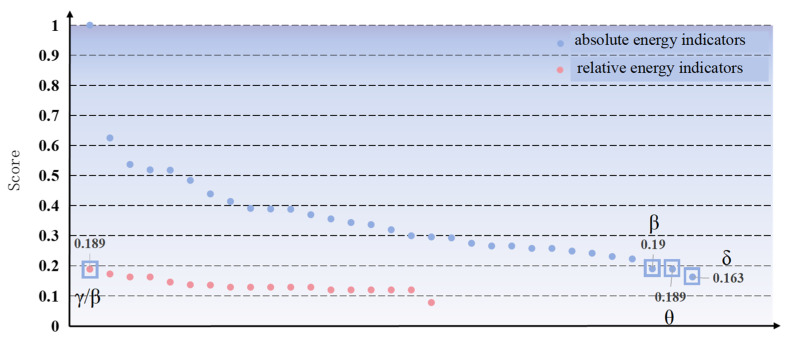
**Scores for various types of indicators.** The indicator score is the average value of normalized classification accuracy under different classification methods, which represents the performance of the indicator. The closer the score is to 1, the better the performance of the indicator.

To evaluate hypothesis H2, a comparison of the scores of absolute energy indicators and relative indicators is presented in Figure 8. Notably, all of the absolute energy indicators were consistently ranked higher than the relative indicators, except for indicators 1 and 2.

Furthermore, to ascertain whether there exists a significant disparity between the absolute energy indicator scores and the relative energy indicator scores, statistical tests were conducted on the two sets of data. Initially, Levene’s test for homogeneity of variances was executed on the samples to assess the extent of deviation of each group’s observations from their respective group means. The resulting *p*-value was 0.003254042167283171, indicating that the variances of the two data sets were not homogeneous. Given the uneven variance and sample size between the two data sets, the Welch’s *t*-test was then carried out. The findings revealed a t-statistic value of 7.45561541056084 and a *p*-value of 1.6265354752083185 × 10^−8^, demonstrating a significant difference between the two data sets, as *p* < 0.05. This result supports the validity of H2, indicating that absolute energy is more effective than relative energy in reflecting the state of controllers under different workloads.

### 3.4. Results of Application

**Figure 9:**
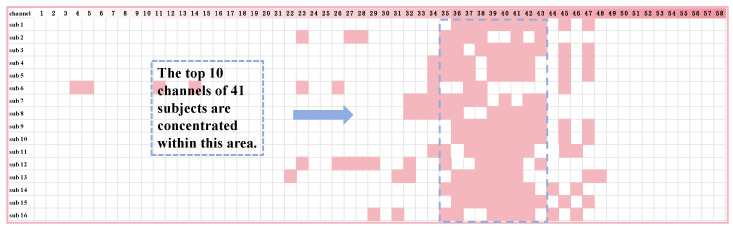
The output of the mRMR algorithm is the top 10 channels in which each subject performed better, and each subject’s channel was colored as shown above.

To identify channels most relevant to the workload of controllers, the mRMR algorithm was applied, with the target variable being workload. Among the 49 indicators, the indicator β + θ + α + γ demonstrated the highest accuracy, and the results of mRMR based on this indicator are detailed in Figure 9, which displays the results of the channel screening, revealing the top 10 ranked channels for the 16 subjects.

The subsequent analysis revealed that each subject corresponded to a different channel ranking, suggesting that the best combination of channels cannot be directly determined using this algorithm. However, it is worth noting that some channels appeared frequently across subjects, suggesting that while a universally optimal channel combination may not exist, it is feasible to identify channel combinations that perform well for the majority of subjects.

To address this issue, the occurrence of each channel was counted, and the results are presented in the Table 5. CP6 and TP5 appeared 14 times, while CP5 and CP2 appeared 13 times, accounting for 75% or more of the total number of subjects.

**Figure 10:**
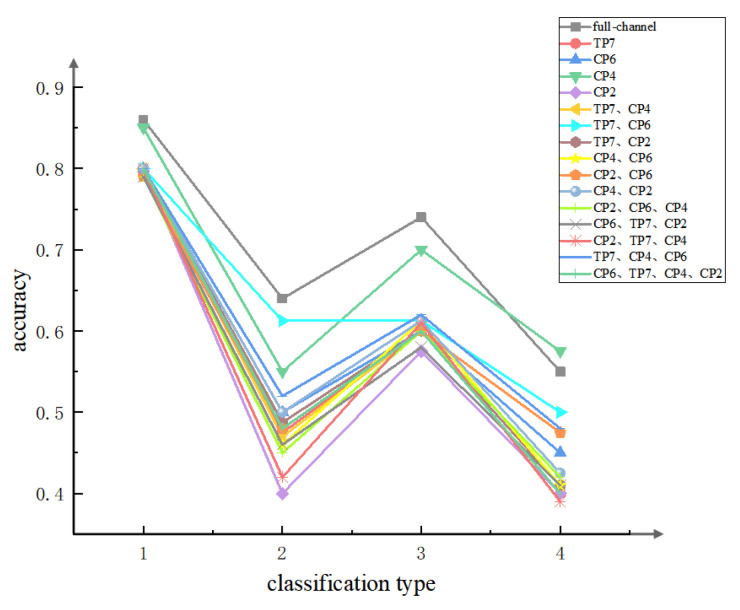
**Comparison of channel accuracy.** Based on Indicator 49, the controller workload is detected using different channels and combinations of channels. The labels are categorized corresponding to the above, where the detection accuracy of all channels is the highest regardless of the categorization method.

Based on these findings, the study organized and combined the mentioned channels and classified the data accordingly using the method described previously. This comprehensive analysis considered a total of 15 combinations, including single-channel, dual-channel, triple-channel, and quad-channel configurations; the detection accuracy of various channel combinations is shown in Figure 10. Notably, the combination of channels 40 and 41 demonstrated a notably high classification accuracy, reaching up to 80% of the full-channel accuracy regardless of the classification mode.

Among all combinations, CP4 exhibits the best performance. In resting/working and low/medium–high load scenes, the classification accuracy reaches up to 95% of the full channel. Even in low/medium–high load scenarios, the accuracy slightly drops but still achieves up to 85.93% of the full channel. Notably, in quadruple classification, the single-channel classification accuracy of channel 38 exceeds the full-channel accuracy, reaching 0.575.

This observation highlights that the number of channels does not directly correlate with classification accuracy. Notably, the detection accuracy by only one channel, CP4, surpasses that of all channel combinations except for the full-channel and the TP7 and CP6 combination under different classification methods. Overall, the combination of Indicator 49 and CP4 demonstrates both convenience and accuracy in detecting the workload of controllers.

## 4. Discussion

### 4.1. Comparison between Indicators

In exploring the application of EEG signals in assessing the workload of controllers, this study initially selects EEG power spectrum indicators associated with delta waves, theta waves, alpha waves, and beta waves. Subsequently, a new set of EEG power spectrum indicators related to gamma waves is proposed. A control simulation experiment is designed to collect experimental data, validating the correlation between these indicators and workload.

The results reveal that the classification accuracy of indicator15 (δ + β + θ + α + γ) surpasses that of indicator5 (δ + β + θ + α), and the classification accuracy of indicator14 (γ absolute energy) exceeds that of the delta, theta, alpha, and beta waves. Moreover, absolute energy proves to better reflect controllers’ workload compared to relative energy. Hence, there is a proposition that gamma waves can be utilized to detect controller workload, along with the novel indicators of absolute energy, to test this hypothesis. Notably, among the 14 groups of indicators, those including gamma waves demonstrate higher classification accuracy compared to those without gamma waves.

**Figure 11:**
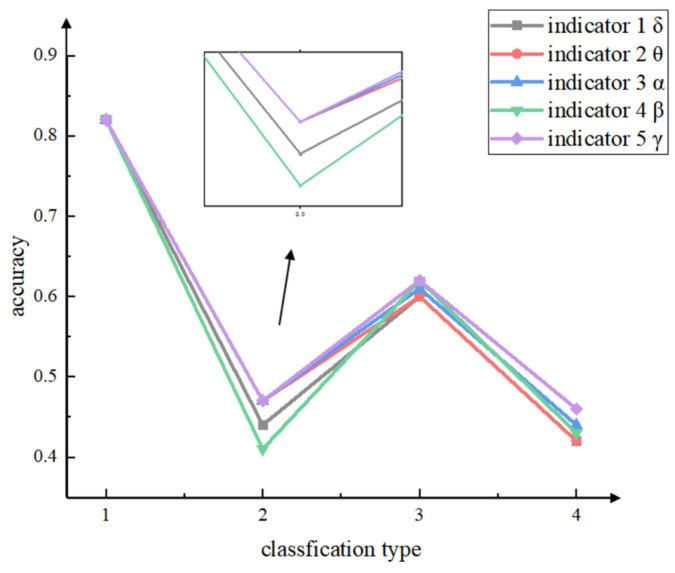
**For comparison of absolute energy indicators across five bands.** Regardless of the classification method utilized, the absolute energy of gamma outperforms the absolute energy of delta, theta, alpha, and beta in terms of accuracy.

From Figure 11, regarding the absolute energy of delta, theta, alpha, and beta waves, the findings of this study align with existing research results [40]. The inherent energy of these waves effectively detects controllers’ workload, achieving a classification accuracy of 80% or higher in distinguishing between resting and working states. Furthermore, they also produce satisfactory results in triple classification and quadruple classification. However, regardless of the classification method utilized, the absolute energy of gamma waves introduced in this paper consistently outperforms the aforementioned indicators in terms of accuracy. In line with previous research [30], this study reinforces the effectiveness of relative energy indicators, specifically θ/β, α/β, (α + θ)/β, and (α + θ)/(α + β), for evaluating the workload of controllers. These indicators displayed a classification accuracy of over 80% in distinguishing between resting and working states, as well as yielding satisfactory results in triple and quadruple classifications. Nonetheless, as shown in Table 6, their accuracies consistently lag behind the relative energy of γ/β introduced in this paper, regardless of the classification mode employed. In conclusion, these results demonstrate that utilizing EEG gamma wave-associated indicators can offer valuable insights into analyzing workload levels among air traffic controllers.

### 4.2. Application of Research Results

A thorough analysis of 49 indicators using four different classification methods in this study revealed variability in the ranking of indicator accuracy across the methods, suggesting a correlation between classification methods and accuracy. Thus, careful selection of the most appropriate indicators and classification methods is crucial when utilizing EEG for workload assessment.

Significantly, regardless of the classification method employed, indicator 49 (β + θ + α + γ) was robust in different categorization methods and consistently demonstrated the highest precision, indicating strong generalizability. This finding aligns with existing research [49], emphasizing the efficacy of combined frequency rhythms over individual rhythms for various purposes. Additionally, the EEG features related to gamma waves introduced in this study prove to be highly suitable for detecting mental workload in controllers compared to conventional features. In summary, the findings of this paper can contribute to the more accurate and convenient detection of the workload of controllers.

### 4.3. Contributions and Limitations

The study confirmed the effectiveness of the indicator β + θ + α + γ in assessing controller workload, with a recognition rate of over 95% for single channel usage, which enhances both convenience and accuracy in practical applications. Furthermore, the research illustrates the significance of utilizing EEG gamma wave-associated indicators to gain valuable insights into air traffic controllers’ workload levels.

Notably, during the verification process, an unexpected discovery was made regarding the higher accuracy of most absolute energy indicators compared to relative energy indicators. However, due to time constraints, this finding was not extensively investigated. Therefore, researchers are encouraged to further explore this conclusion by proposing additional absolute and relative energy indicators based on this hypothesis for experimental verification.

## 5. Conclusions

In this study, novel power spectrum indicators based on gamma waves were proposed for identifying controller workload in an EEG-based system. The results demonstrate that the indicators related to gamma waves presented in this paper exhibit higher accuracies compared to traditional indicators. The following was found:

(1) β + θ + α + γ demonstrates the highest accuracy regardless of the classification method. This indicator allows for the feasible use of a single channel in detecting controller workload, with a recognition rate exceeding 95% of the full channel. This meets the practical application requirements for both convenience and accuracy.
(2) The best channel or channel combination varies from one research subject to another; in other words, the best-performing channel varies from person to person.
(3) Classification methods have a significant impact on classification accuracy. In this study, two triple classification methods were used, and the accuracy of low/medium–high/low load is usually higher than low/high/medium–low load.
(4) Different controllers perceive the workload of special scenes differently, and one of the reasons for this phenomenon might be the variations in work experience.
(5) The detection accuracy of most absolute energy indicators is higher than that of relative energy indicators.
(6) The number of channels is not proportional to the classification accuracy; in other words, it is not the case that the greater the number of channels, the higher the accuracy of detection.

## Figures and Tables

**Table 1:** Control scene selection in experiment.

Scene	Exercise	Workload	Air Traffic Flow
Scene 0	/	0	0
Scene 1	PX25-12P02	lower load	6
Scene 2	PX25-17M01	higher load	17
Scene 3	TWR401-07-1	overload	30
Scene 4	PX25-17M01	abnormal situation	17

**Table 2:** Proposal of indicators.

Indicator1	δ Absolute Energy	Indicator14	γ Absolute Energy
indicator2	θ absolute energy	indicator15	δ + β + θ + α + γ
indicator3	α absolute energy	indicator16	γ/δ
indicator4	β absolute energy	indicator17	γ/θ
indicator5	δ + β + θ + α	indicator18	γ/α
indicator6	δ/(δ + β + θ + α)	indicator19	γ/β
indicator7	θ/(δ + β + θ + α)	indicator20	γ/(α + β)
indicator8	α/(δ + β + θ + α)	indicator21	γ/(θ + β)
indicator9	β/(δ + β + θ + α)	indicator22	γ/(θ + α)
indicator10	θ/β	indicator23	γ/(θ + β + α)
indicator11	α/β	indicator24	γ/(δ + β + θ + α)
indicator12	(α + θ)/β	indicator25	γ/(δ + β + θ + α + γ)
indicator13	(α + θ)/(α + β)		

**Table 3:** S-W test of NASA-TXL data.

Variable	Sample Size	Mean Value	Standard Deviation	S-W Statistics	*p*-Value
Scene 4	16	42.5	7.598	0.9289878	0.2349399
Scene 3	16	55.835	8.644	0.9659469	0.7694572
Scene 1	16	40.835	13.045	0.95286422	0.5363789
Scene 2	16	46.67	11.842	0.90147995	0.0848791

**Table 4:** Normalized data.

Indicator	Average Value	Indicator	Average Value	Indicator	Average Value
β + θ + α + γ	1.000	δ + θ	0.300	γ/(θ + α)	0.163
δ + β + θ + α + γ	0.625	γ	0.296	δ	0.163
δ + θ + α + γ	0.537	δ + β + α	0.275	α/β	0.146
δ + β + θ + γ	0.519	β + α + γ	0.266	γ/θ	0.137
δ + β + θ + α	0.518	β + θ + γ	0.266	θ/β	0.136
δ + β + α + γ	0.484	β + α	0.258	δ/(δ + β + θ + α)	0.129
θ + α + γ	0.439	α + γ	0.258	α/(δ + β + θ + α)	0.129
δ + θ + α	0.414	θ + γ	0.249	γ/δ	0.129
δ + β + θ	0.391	α	0.242	γ/(θ + β)	0.129
β + θ + α	0.389	β + θ	0.231	γ/(θ + β + α)	0.129
δ + α + γ	0.371	β + γ	0.223	θ/(δ + β + θ + α)	0.120
δ + γ	0.388	β	0.190	β/(δ + β + θ + α)	0.120
δ + α	0.370	γ/β	0.189	γ/(α + β)	0.120
δ + β + γ	0.361	θ	0.189	γ/(δ + β + θ + α)	0.120
δ + θ + γ	0.356	γ/α	0.173	γ(δ + β + θ + α + γ)	0.120
δ + β	0.344	(α + θ)/(α + β)	0.163	(α + θ)/β	0.078
θ + α	0.337				

**Table 5:** Channel occurrences.

Channel by Number	Channel by Name	Occurrences	Frequency
40	CP6	14	87.5%
41	TP7	14	87.5%
39	CP5	13	81.25%
36	CP2	13	81.25%
37	CP3	12	75%
38	CP4	12	75%
43	Pz	12	75%
35	CP1	10	62.5%

**Table 6:** Comparison of relative energy indicators.

Indicator	Resting/Working	0/High/Medium–Low Load	0/Medium–High/Low Load	0/Medium/High/Low Load
indicator10 θ/β	0.82	0.43	0.6	0.41
indicator11 α/β	0.82	0.4	0.6	0.43
indicator12 (α + θ)/β	0.81	0.42	0.6	0.41
indicator13 (α + θ)/(α + β)	0.82	0.42	0.6	0.43
indicator19 γ/β	0.82	0.45	0.6	0.43

## Data Availability

The data presented in this study are available upon request from the corresponding author. The data are not publicly available due to confidentiality issues.
